# Supplementary material for: Getting More Out of Biomedical Documents with GATE's Full Lifecycle Open Source Text Analytics
Source: PLoS Comput Biol. 2013 Feb 7;9(2):e1002854. doi: 10.1371/journal.pcbi.1002854 (PMC3567135; doi:10.1371/journal.pcbi.1002854)
Supplement: Dataset S1 — GATE software. Dataset S1 bundled with this paper contains a distribution of GATE (or see http://gate.ac.uk/download/). (TGZ) [file pcbi.1002854.s001.tgz › plos-gate/plugins/Gazetteer_LKB/doc/gate_compatibility.html]

The Large KB gazetteer - GATE Compatibility


---

Last Published: 2009-10-17

---

##### Large KB Gazetteer

- Overview
- Usage
- Q & A
- **GATE Compatibility**
- GATE Gazetteer Feature Comparison
- Semantic Enrichment PR
- Samples

##### Developer Documentation

- Dictionary Lifecycle

## GATE 5.0 Compatibility

You can modify a GATE 5.0 installation to make it compatible with the large KB gazetteer. Delete the following libraries from the *lib* folder of GATE - openrdf-util.jar, openrdf-model.jar, sesame.jar and owlim.jar. Note that that will disable the Ontology plugin, all plugins that depend on it and all plugins that use the Sesame 1 API.

## Compatibility with other CREOLE plugins

From GATE 5.1 onwards, the plugins can load any RDF API. The large KB gazetteer uses the Sesame 2 API and may not work correctly if plugins that use the Sesame 1 API are loaded.

---

©
2009
Ontotext AD

---
